# The Respiratory Protection Effectiveness Clinical Trial (ResPECT): a cluster-randomized comparison of respirator and medical mask effectiveness against respiratory infections in healthcare personnel

**DOI:** 10.1186/s12879-016-1494-2

**Published:** 2016-06-02

**Authors:** Lewis J. Radonovich, Mary T. Bessesen, Derek A. Cummings, Aaron Eagan, Charlotte Gaydos, Cynthia Gibert, Geoffrey J. Gorse, Ann-Christine Nyquist, Nicholas G. Reich, Maria Rodrigues-Barradas, Connie Savor-Price, Ronald E. Shaffer, Michael S. Simberkoff, Trish M. Perl

**Affiliations:** U.S. Department of Veterans Affairs, National Center for Occupational Health and Infection Control, 1601 SW Archer Road, Mailstop 151E, Gainesville, FL 32608 USA; Veterans Affairs Eastern Colorado Healthcare System, Denver, CO USA; Johns Hopkins School of Medicine, Baltimore, MD USA; Veterans Affairs Medical Center and George Washington University School of Medical and Health Sciences, Washington, DC USA; Veterans Affairs St. Louis Healthcare System and Saint Louis University School of Medicine, St. Louis, MO USA; Children’s Hospital Colorado, Aurora, CO USA; Department of Biostatistics and Epidemiology, University of Massachusetts, Amherst, MA USA; Michael E. DeBakey VAMC and Baylor College of Medicine, Houston, Texas USA; Denver Health, Denver, CO USA; National Personal Protective Technology Laboratory, National Institute for Occupational Safety and Health, Pittsburgh, PA USA; Veterans Affairs New York Harbor Healthcare System, New York, NY USA; University of Colorado School of Medicine, Denver, CO USA; Johns Hopkins Bloomberg School of Public Health, Baltimore, MD USA; University of Florida, Gainesville, Florida USA

**Keywords:** Respirators, Masks, Healthcare personnel, Influenza, Respiratory infections

## Abstract

**Background:**

Although N95 filtering facepiece respirators and medical masks are commonly used for protection against respiratory infections in healthcare settings, more clinical evidence is needed to understand the optimal settings and exposure circumstances for healthcare personnel to use these devices. A lack of clinically germane research has led to equivocal, and occasionally conflicting, healthcare respiratory protection recommendations from public health organizations, professional societies, and experts.

**Methods:**

The Respiratory Protection Effectiveness Clinical Trial (ResPECT) is a prospective comparison of respiratory protective equipment to be conducted at multiple U.S. study sites. Healthcare personnel who work in outpatient settings will be cluster-randomized to wear N95 respirators or medical masks for protection against infections during respiratory virus season. Outcome measures will include laboratory-confirmed viral respiratory infections, acute respiratory illness, and influenza-like illness. Participant exposures to patients, coworkers, and others with symptoms and signs of respiratory infection, both within and beyond the workplace, will be recorded in daily diaries. Adherence to study protocols will be monitored by the study team.

**Discussion:**

ResPECT is designed to better understand the extent to which N95s and MMs reduce clinical illness among healthcare personnel. A fully successful study would produce clinically relevant results that help clinician-leaders make reasoned decisions about protection of healthcare personnel against occupationally acquired respiratory infections and prevention of spread within healthcare systems.

**Trial registration:**

The trial is registered at clinicaltrials.gov, number NCT01249625 (11/29/2010).

## Background

Healthcare personnel (HCP) are exposed to respiratory pathogens in many clinical settings [1]. Infected HCP may spread infection to their patients [2–5] or coworkers [3–6], to family members [4, 7], or to other community members [4, 8]. Respiratory viral infections among healthcare workers can negatively impact delivery of healthcare services [9–11].

United States national guidelines call for modes of transmission to dictate infection control measures [3]. For most human respiratory viruses, the precise mode(s) of person-to-person transmission is incompletely understood [12, 13]. The predominant mode of transmission for some human respiratory pathogens, such as influenza virus, respiratory syncytial virus, and coronavirus is believed to be *droplet transmission. Airborne transmission* plays a role with some human respiratory pathogens via small aerosol particles, often called droplet nuclei [3]. Airborne transmission is the predominant mode of transmission for Mycobacterium tuberculosis [3, 14] and recent evidence has suggested a larger role than previously thought for Influenza A and B viruses [15, 16].

Disposable respiratory protective devices (RPD) that fit tightly to the wearer’s face, sometimes called air-purifying respirators or filtering facepiece respirators, are primarily designed to *protect the wearer* against infection spread by ill patients. N95 filtering facepiece respirators (commonly known as “N95 respirators”) are one type of RPD capable, with proper facial fit and usage, of reducing inhalation of airborne particulates by a factor of 10 or greater [17]. Medical masks (MM), typically called surgical masks in operative settings, are primarily devised to *protect patients* against infection spread by the wearer [18]. Both types of devices also serve as a physical barrier keeping sprays and splashes of infectious materials and contaminated hands and objects away from oronasal region of wearer. Although RPD and MM are capable of filtering particulates [19], RPD are designed to filter smaller particulates that may remain airborne for long periods. A tight seal between the respirator and the wearer’s face is designed to prevent leakage of particulates, a feature not provided by loose-fitting MM. The U.S. Department of Labor’s Occupational Safety and Health Administration (OSHA) requires employers to ensure each HCP, who may be exposed to airborne-transmissible infections in the workplace, receives an RPD with an adequate respirator-to-face seal that is determined during a mandated annual “fit-test”.

However, evidence is inconclusive that RPD are better than MM at protecting HCPs from respiratory infections in clinical settings [20–25], despite tight-fitting RPD produced by manufacturers, with higher levels of exposure reduction validated by numerous laboratory studies [19, 26–28], and the use of a complete respiratory protection program (e.g., training, initial and annual fit test) as defined by OSHA to protect HCP. Intuitively, RPD should better protect HCP against airborne infections than MM, but objective evidence has not validated this supposition. One possibility that may explain this discrepancy between expectations and observations is pragmatic: HCP, in general, do not tolerate N95 respirators as well as medical masks [29, 30], perhaps prompting them to remove respirators more frequently and/or for longer periods, increasing the likelihood of exposure to infections. Models have shown that 25 % or more non-wear time during exposure negates any significant differences in protective ability between types of RPDs [17, 31]. Given the difficulty with HCP adherence to guidelines [4] and general dissatisfaction [4, 32–34] with RPD, medical masks worn more consistently may provide similar levels of reduction in respiratory viral disease transmission as N95 respirators.

This key gap in knowledge has contributed to discrepant clinical and public health recommendations about respiratory protection for HCP [35, 36]. Needed are additional well-designed clinical trials conducted in patient-care settings during outbreaks of respiratory infections. The following is an abridged version of the full research protocol for the Respiratory Protection Effectiveness Clinical Trial (ResPECT).

### Objective

To compare the effectiveness of N95 respirators and medical masks at protecting HCP from acquiring viral respiratory illnesses in the workplace.

### Hypotheses

Null Hypothesis: The incidence of laboratory confirmed influenza (primary), influenza-like illness (ILI), acute respiratory illness (ARI) and other respiratory infections will not be different between HCPs who practice 2007 guidelines (medical masks) or 2009 guidelines (N95 respirators).

Alternative Hypothesis: The incidence of laboratory confirmed influenza (primary), influenza-like illness (ILI), acute respiratory illness (ARI) and other respiratory infections will be different between HCPs who practice the CDC’s 2007 guidelines for influenza protection (medical masks) versus 2009 guidelines for influenza protection (N95 respirators).

## Methods

### General overview

ResPECT is a prospective comparison of respiratory protective equipment to be conducted at multiple, geographically distributed U.S. study sites. HCP who work in outpatient settings will be cluster-randomized to wear N95 respirators [37] or MM [38] for protection against infections during respiratory virus season, the “intervention” period.

The null hypothesis assumes N95 and MM intervention groups will have no differences in outcomes, including (1) laboratory confirmed influenza or (2) influenza-like illness (ILI), (3) acute respiratory illness (ARI), and (4) laboratory confirmed respiratory illness (LCRI). The alternative hypothesis asserts the incidence of at least one outcome would be different between intervention groups.

Because respiratory virus season varies year-to-year in onset, severity, and duration, multiple season-years of the study will be necessary to account for expected variance and optimally generalize the resulting knowledge. The beginning of each season’s data collection will be independently determined for each study site using an epidemiologic predictive tool designed for ResPECT to capture the largest possible number of respiratory infections. These data will be collected for twelve weeks each season.

Participant exposures to patients, coworkers, and others with symptoms and signs of respiratory infection, both within and beyond the workplace, will be recorded in daily diaries. Adherence to study protocols will be measured by the study team at each site. Since periodic changes in infection control guidance and practice may occur over the study years, participants will be expected to adhere to the most up-to-date guidance issued by the Centers for Disease Control and Prevention (CDC) and local policies at each study institution, at a minimum. For example, a participant randomized to the MM arm will be expected to don an N95 when participating in an aerosol-generating procedure, assuming no further changes in pertinent national guidance [39].

### Participants and setting

The study participants will be recruited from outpatient settings where patients are relatively likely to present with symptoms and signs of acute respiratory infection. Participants will be eligible to enroll for multiple study seasons, yet each will be provided with informed consent and complete enrollment procedures prior to each study season.

Clinical Study sites will be distributed geographically:

1. Veterans Affairs New York Harbor Healthcare System; New York, NY
2. Johns Hopkins Health System and University; Baltimore, MD;
3. Washington DC Veterans Affairs Healthcare System; Washington, DC;
4. Veterans Affairs Eastern Colorado Healthcare System; Denver, CO;
5. Denver Health; Denver, CO
6. Childrens’ Hospital Colorado; Aurora, CO
7. Michael E. DeBakey Veterans Affairs Medical Center; Houston, TX

### Inclusion criteria

- Clinical study site leadership agree to have one or more staff participate
- Participant meets the definition of “healthcare personnel”
- Provides healthcare to patients and/or
- Routinely positions herself/himself within 6 feet of patients (“close contact”) and
- Is a full-time employee (average of ≥ 24 hours/week) working 75 % of the time at a study site and not employed at another location where the study is being conducted
- Participant is able to read and sign informed consent
- Participant agrees to all requirements of the protocol, including fit-testing and diary-keeping
- Participant is age 18 years or greater
- Participant passes fit-testing for at least one of the study respirator models and, if assigned to the respirator arm, agrees to use that model for the seasonal study period.

### Exclusion criteria

1. Participant self-identifies as having severe heart, lung, neurological or other systemic disease that one or more Investigator believes may preclude safe participation.
2. Participant is known to not tolerate wearing respiratory protective equipment for any period.
3. Participant has facial hair adornments or other anatomic features that preclude respirator Occupational Safety and Health Administration (OSHA)-compliant fit-testing or proper fit during the seasonal data collection period.
4. Participant is advised by a study site occupational health clinician (or other qualified clinician) to not wear the same or similar respirator or medical mask models used in ResPECT.
5. Participant self-identifies as pregnant in the third trimester during the seasonal study period.
6. Participant is working in more than one ResPECT study sites during the seasonal study period.
7. Participant is working less than 24 hours per week during the seasonal study period at a ResPECT study site.
8. Participant is working less than 75 % of the seasonal study period at a ResPECT study site.
9. Participant is a previous ResPECT study participant who does not consent to have her/his data linked from a previous viral respiratory season(s).
10. In the opinion of a principal investigator, participant may not be able to join the trial for any other reason.

### Interventions

#### N95 respirators and medical masks

Participants will be cluster-randomized to one of the following N95 respirators or MM models, selected because they are commonly used in U.S. medical facilities, including the ResPECT study sites. Participants who participate in more than one of the study years will be cluster-randomized anew each year.

N95 Respirators:

1. 3 M Corporation 1860, 1860S, and 1870 models (St Paul, MN) or
2. Kimberly Clark Technol Fluidshield PFR95-270, PFR95-274 (Dallas, TX).

Medical Masks:

1. Precept 15320 or
2. Kimberly Clark Technol Fluidshield 47107.

Respirator Fit

All subjects participating in the study will be required to pass an OSHA-accepted respirator fit test for the N95 respirator model(s) available at the study site. No fit testing of medical masks will be performed as these devices are not designed to be tight-fitting to the face and studies [19, 20] have shown that their fit capabilities are generally low.

Filter Performance

Although medical masks are loose-fitting, they create a physical barrier that helps prevent splashes and sprays from reaching the wearer’s mucous membranes. In addition to passage *around* the mask, some of the small particle aerosols are able to pass *through* the mask’s filter media. Therefore, in addition to RPDs, filtration testing was done on medical masks prior to enrollment of subjects to ensure consistency between models across study locations. The filtration performance of the N95 respirators and medical mask models in the study were tested in a manner similar to that used by the National Institute for Occupational Safety and Health (NIOSH). Devices were attached to a test fixture and placed in a TSI 8130 automated filter tester operated with an air flow rate of 85 liters per minute. The TSI 8130 uses a photometer to measure the flux of light scattering from aerosol particles. Polydispersed particles (mass median diameter of ~0.3 microns) were generated from a 2 % NaCl solution and passed through each device being tested for 1 min. Each test was repeated 3 times with a fresh N95 respirator or medical mask. To be certified as an N95 respirator, filter penetration needs to be less than 5 % (or 95 % efficient). As shown in Table 1, the average penetration percentages for the NIOSH certified N95 respirators were an order of magnitude lower than those of medical masks, which are not NIOSH certified. Filter results between N95 respirator models and between medical mask models were comparable.

**Table 1:** Study RPD particle penetration and airflow resistance

Device name	% penetration^a^	Resistance (mmH_2_O)^a^
N95 respirators		
3 M 1860/1860S	0.7	8.9
3 M 1870	0.3	9.6
Kimberly Clark Technol Fluidshield PFR95-270/274	1.4	11.7
Medical Masks		
Precept 15320	12.9	4.1
Kimberly Clark Technol Fluidshield 47107	10.3	4.5

^a^Average value from 3 replicate measurements

Filter airflow resistance was measured simultaneously using the TSI 8130. As filter airflow resistance increases, more energy expenditure is required for ventilation during device wear and the greater potential for perception of discomfort [40]. The medical mask models selected for this study have filter airflow resistance levels about half of that of the N95 respirators. However, one study [40] found that subjective and physiological responses were not different among subjects exercising while wearing devices purposely made with different filter airflow resistance levels (3 mm H_2_O, 6 mm H_2_O, and 9 mm H_2_O) in the range similar to those of the devices in this study (Table 1).

#### Adherence to intervention arm and hand hygiene performance

Participants will be instructed to don a new N95/MM with each patient interaction, every time a participant encounter occurs within 6 feet of a patient who has suspected or confirmed respiratory infection. Hand hygiene will be recommended to all participants in accordance with CDC guidelines [41] and policies at each study institution. Trained research assistants will observe participants during study periods to assess adherence to their assigned intervention arms and hand hygiene. A portable computer equipped with data recording software (HandyAudit; Toronto, Canada) will be used to document adherence. Participants will be expected to complete surveys about their attitudes and opinions concerning personal protective equipment before and after each seasonal study period.

#### Estimation of exposure

During the twelve week data collection period each year, participants will self-document (a) perceived occupational exposures to patients or coworkers who have symptoms or signs of respiratory infection, (b) perceived non-occupational exposures to persons who have symptoms or signs of respiratory infection, (c) use of personal protective equipment, and (d) personal symptoms or signs of respiratory illness.

### Study outcome definitions

#### Collection of specimens

Anterior nasal and pharyngeal swabs [42–45] [FLOQSwabs UTM (99–08024), Diagnostic Hybrids; Athens, OH] will be collected by research assistants when symptomatic with study defined respiratory symptoms, as well as two, randomized asymptomatic swabs during each seasonal study period. Swabs will be collected when (a) participants self-report respiratory symptoms within a 24 h period, and again if participants remain symptomatic after 7 days; and (b) randomly, on all participants, twice during the active intervention period.

***The Primary Outcome Measures*** will be the incidence of:

Laboratory-confirmed influenza (LCI) A or B infection in participants, defined as a) detection of influenza virus by reverse-transcription polymerase chain reaction (RT-PCR) in an upper respiratory specimen swab collected within seven days of symptom onset, or b) influenza seroconversion defined as at least a 4-fold rise in hemagglutination inhibition antibody (HAI) titers to influenza A or B virus from the pre- to post-season serological samples that is not deemed attributable to vaccine.

***The Secondary Outcome Measures*** will be the incidence of:

1. *Acute Respiratory Illness (ARI)* defined as the occurrence of one sign or two symptoms (Table 2) without laboratory confirmation.

**Table 2:** Case definition of acute respiratory illness used in the ResPECT study

Signs	
Fever (T > 37.8 °C)	
Tachypnea (Respiratory Rate ≥ 25)	
Coryza	
Lymphadenopathy	
Symptoms	
Vomiting/Nausea	
Diarrhea	
Cough	
Sputum production	
Fatigue	
Malaise	
Headache	
Sore throat	
Dyspnea	
Chills	
Sweats	
Arthralgias/Myalgias/Body Aches	
Other gastrointestinal symptoms	

*Acute respiratory illness (ARI) is defined as: The presence of one sign(s) OR two symptom(s), as listed. Reported signs or symptoms must represent a *change from baseline*
2. *Laboratory Confirmed Respiratory Illness (LCRI)* defined as self-reported ARI plus the presence of one or more RT-PCR confirmed infectious pathogens (Table 3) in a specimen collected from the upper respiratory tract and/or a clinically significant rise in pre- and post-season serum antibody titers to influenza A or B virus.

**Table 3:** Potential influenza like illness pathogens

Influenza A	
Influenza B	
Respiratory syncytial virus type A	
Respiratory syncytial virus type B	
Parainfluenza virus type 1	
Parainfluenza virus type 2	
Parainfluenza virus type 3	
Parainfluenza virus type 4 (a)	
Parainfluenza virus type 4 (b)	
Human Metapneumovirus	
Adenoviruses	
Coronavirus OC43	
Coronavirus NL63	
Coronavirus 229E	
Coronavirus HKU1	
Human Rhinovirus	
Cocksackie/echoviruses	
Bocavirus
3. *Influenza-Like Illness (ILI)* defined as temperature of 100 °F [37.8 °C] or greater plus cough and/or a sore throat, with or without laboratory confirmation.

The incidence rate ratios between participants randomly assigned to wear N95 respirators or medical masks will be estimated for each of the primary and secondary outcomes.

#### Outcome determination, validation, and adjudication

Investigators will be paired and provided with blinded information about clinical and laboratory data to determine if a participant meets a primary or secondary outcome. If the paired investigators do not agree, a principal investigator will adjudicate the outcome.

### Laboratory methods

#### Respiratory pathogen identification

Assays will be performed at Johns Hopkins University.

Collected respiratory specimens will be stored at −80 °C until analyzed using multiplex PCR (PLEX-ID, Abbott Labs, Chicago IL). Automated extraction of nucleic acid (NA) from respiratory specimens will be performed utilizing NorDiag’s Arrow instrument and the Magna Pure robotic system (Roche Indianapolis, IN) per manufacturer instructions. Each extraction run will include a quality control (NATrol Respiratory Validation Panel 3, Zeptometrix Inc., Buffalo NY); runs with control failures will be repeated. Purified NA will be amplified via RT-PCR using a broad respiratory virus identification kit (PLEX-ID RVS 3.0, Abbott Molecular, Des Plaines, IL). Desalting of RT-PCR product and electrospray mass spectrometry-based NA analysis will be performed on the PLEX-ID analyzer instrument. If funding is sufficient, samples will also be assayed by RT-PCR for *Bordetella pertussis, Mycoplasma pneumoniae,* and for *Chlamydophila pneumoniae.*

#### Serologic testing

Each study season, blood samples will be collected twice from each participant; one sample will be collected within two weeks of the beginning of the intervention period and a second within two weeks of the end of the intervention period. Hemagglutination inhibition (HAI) antibody assays will be performed on serum for influenza A and B virus strains, dependent on the antigens in each annual trivalent vaccine using standard methods [46, 47]. In brief, serial 2-fold dilutions of serum samples will be incubated with 8 hemagglutinin units of influenza antigen and a turkey red blood cell suspension. The serum HAI antibody titer will be defined as the dilution factor of the highest serum dilution that completely inhibits agglutination of turkey red blood cells in the presence of type-specific hemagglutinin antigen. Assays will be performed at the immunology core lab for the study at the VA Saint Louis Veterans Affairs Healthcare System.

### Statistical methods

#### Randomization

To optimize compliance and generalizability, a cluster-randomized design will be utilized. All participants working in the same clinical unit will be assigned to wear the same respiratory protective equipment (i.e., an N95 or MM) during patient interactions for the entire 12 week seasonal study period. Clusters will be pair-matched within each study site based on the characteristics of each clinical cluster, including the (a) number of participants (b) occupational location, such as an emergency department, urgent care or primary care, (c) patient population served, such as children or adults, and (d) requirements for participants to wear additional protective equipment, such as goggles donned by dental hygienists. For each study season, the clinics in each matched pair will be randomly assigned to opposing study arms. For matched pairs participating in multiple study seasons, random sequences of arm assignments will ensure each is assigned to both study arms during the multi-year study. Each study season, an individual not involved in the study implementation and data analyses will perform the randomization scheme for each study site, using a random number generator in Microsoft Excel. The principal investigators will be blinded to the randomization scheme prior to assignment.

#### Statistical analyses

Incidence rates of LCI, ARI, ILI, and LCRI among cluster-randomized participants will be compared. The relationships between incidence of clinically diagnosed and laboratory-confirmed illnesses will be analyzed with attention to potential confounders, such as participants’ demographics, study arm compliance, attitudes and opinions about infection control, receipt of influenza vaccination, and infectious exposures within and beyond the workplace. Standard statistics will describe baseline characteristics and follow-up measures, summarized by treatment arm and stratified by study site.

To assess the primary outcome, a logistic regression model will be fit using a dichotomous variable to indicate whether a participant became infected with a respiratory pathogen. The odds of infection between the two treatment groups will be reported with a 95 % confidence interval. For secondary outcomes, Poisson log-linear mixed effects regression models will assess the difference in seasonal respiratory infection rates between intervention groups. Cluster- and individual-level random effects will be considered to account for clustered observations. Additional covariates may be added to the models to adjust for confounding.

#### Missing data

Participants will be encouraged to complete the study. Those who withdraw from an intervention arm will be encouraged to complete follow-up laboratory specimen collection. An intent-to-treat analysis, in which all available data on all randomized participants are included, will be used for the primary comparison of interventions. A per-protocol secondary analysis will compare treatment effectiveness, accompanied by a planned sensitivity analysis that accounts for participants from whom researchers were not able to obtain a second serological sample.

#### Sample size and power calculations

To detect a 25 % reduction (i.e., a relative risk of 0.75) in the incidence of laboratory confirmed influenza or laboratory confirmed respiratory illness among participants wearing an N95 respirator, compared to participants wearing a medical mask, ResPECT will need to accumulate approximately 10,024 or 5104 person-seasons of data over four seasons respectively.

Sample size calculations are based on several assumptions about the incidence rate and levels of within-cluster correlation. The attack rate laboratory-confirmed influenza during a single study season is assumed to be 20 % among unvaccinated individuals in the medical mask group. We assume 65 % of our population will be administered a vaccine that is 65 % effective in preventing influenza infection. Vaccine effectiveness at the higher end of published reports (86 % in health care workers) will lead to a reduction in the yearly attack rate to approximately 8.8 %, and effectiveness at the lower end of published reports (51 % in the general population) would lead to an increased yearly attack rate of approximately 13.4 %. Importantly, the anticipated effect on the needed sample size of annual variations in influenza incidence is larger than the expected impact of variation in vaccine effectiveness.

The ResPECT study will need 157 independent clusters with a median size of 16 participants each to achieve 80 % power to detect a relative risk of 0.75 between N95 and surgical masks at preventing laboratory-confirmed influenza infection, with a Type-I error rate of 0.05. The total number of individuals participating each season will need to be approximately 2506, with 10,024 total person-seasons accumulating over the multi-year study. For the secondary outcome of laboratory confirmed respiratory illness, the estimated total number of clinics will need to be 80, the total number of individuals participating each season will need to be 1276, and total person-seasons accumulated need to be 5104 (Table 4) over the multi-year study. The sample size are made using the clusterPower software package for R [48]. Power is estimated using an expected annual attack rate of 12 % {12 % = 0.35*0.2 + 0.65*(1–0.65)*0.2} [13]. This yearly attack rate translates into a 4-year attack rate of 39 % {39 % = 1-(1-(0.35*0.2 + 0.65*0.35*0.2))^4^. Accounting for correlation of outcomes within clusters by assuming the correlation coefficient is 0.1, leads to a design effect of 2.5.

**Table 4:** Sample size and power calculations for primary and secondary outcome

	Laboratory confirmed influenza	Laboratory confirmed respiratory illness
Annual attack rate, Medical Mask group	0.12	0.25
Cumulative 4-year attack rate, Medical Mask group	0.39	0.68
Detectable relative risk	0.75	0.75
Median cluster size	16	16
ICC	0.1	0.1
Total person-seasons of observation	10,024	5104

For scenarios representing the lower and higher ends of anticipated attack rates in the medical mask group, two quantities were calculated (a) the power to detect a relative-risk of 0.75 between the N95 group and the medical mask group and (b) the relative-risk that can be detected with 80 % power (Table 5). For all of these calculations the two-sided Type I error probability is 0.05.

**Table 5:** Power analysis of the sensitivity to the 4-year attack rate

	Low attack rate scenario			High attack rate scenario		
*Outcome*	*Medical mask attack rate*	*Power (RR = 0.75)*	*Detectable RR (80 % Power)*	*Medical mask attack rate*	*Power (RR = 0.75)*	*Detectable RR (80 % Power)*
Primary	0.2	43 %	0.62	0.5	93 %	0.80
Influenza like illness	0.15	33 %	0.56	0.4	82 %	0.76
Acute respiratory illness	0.5	93 %	0.80	0.95	100 %	0.94
Laboratory confirmed respiratory illness	0.3	91 %	0.79	0.7	100 %	0.90

#### Sensitivity analysis

##### Potential outcome analysis for laboratory-confirmed influenza

Some data on the primary outcome may be missing due to participants withdrawing from the study early and missing the second serological sample. To account for the unavoidable uncertainty posed by missing primary outcome data, due to participant withdrawal or loss of follow-up, a sensitivity analysis will be conducted that randomly assigns binary outcomes to participants who did not complete the study. A two-dimensional grid will be created that varies the influenza attack rates among participants who withdraw. Withdrawal attack rates in both arms will be fixed between half and twice the observed attack rates, based on complete data. By varying these two parameters across the grid, and for each combination, the adjusted odds ratio will be calculated by averaging across *n* = 50 imputed datasets for each point on the grid.

##### Analysis of differential withdrawal

The characteristics at the time of randomization for participants without complete follow-up will be examined. To assess the potential biases introduced by differential withdrawal among different N95 respirators, a comparison of withdrawal rates and time to withdrawal will be included as an ancillary analysis to the analyses of the primary and secondary outcomes.

### Ethical approvals

ResPECT will be approved by the institutional review board at each participating study site and the Centers for Disease Control and Prevention, prior to study initiation. (*An unabridged version of the ResPECT protocol was approved by the intitutional review board at each study site and the Centers for Disease Control and Prevention*).

### Data safety monitoring board

A Data Safety Monitoring Board (DSMB) consisting of three or more members will be convened. Members with germane expertise in clinical infectious diseases, epidemiology, and clinical trials will be sought, changing in number and composition to meet study needs. The DSMB will independently monitor interim data, to which the investigators will be blinded, identifying an appropriate times for protocol modification or termination.

### Sponsors

ResPECT is jointly funded by the U.S. Department of Veterans Affairs (Veterans Health Administration) and the U.S. Department of Health and Human Services (Centers for Disease Control and Prevention, National Institute for Occupational Safety and Health, and the Biomedical Advanced Research Project Authority).

## Discussion

Viral respiratory infections cause a wide range of illnesses, varying from mild to severe, in HCPs who may spread infection to their patients, family members, and other community members. Healthcare-associated infections cost $10B annually in U.S [49]. Factors influencing transmission of respiratory infections in healthcare facilities include the population density of ill patients in healthcare settings, the types of exposures within healthcare settings, the administrative and physical structures of healthcare facilities, and intrinsic characteristics of virulence [3]. Measures to prevent transmission within healthcare facilities include HCP vaccination, hand-hygiene, cleaning and disinfection of inanimate surfaces, pre- and post-exposure antiviral chemoprophylaxis, patient isolation, and personal protective equipment [3, 6]. ResPECT is designed to better understand the extent to which PPE, specifically represented by differences in exposure reduction afforded by N95s and MMs, reduces clinical illness among HCPs.

While it may seem that N95 respirators should better protect HCPs than MM against airborne infections in the workplace, this notion has not been validated by objective clinical evidence. Low tolerance to respirator wear among HCPs may prompt more frequent or longer periods of removal, compared to MM, to an extent that the benefits of higher levels of filtration and lower levels of leakage around the facial seal afforded by respirators is offset or subjugated.

Key sources of variability in HCP health outcomes are difficult to control for, even in a rigorously designed clinical study such as ResPECT. For example, the inability to prevent HCP community exposures to respiratory infections and the inherent year-to-year variation of viral respiratory infections provide a challenging setting in which to evaluate the effectiveness of personal protective equipment. While community-acquired infections may pose a significant source of exposure for HCPs, this type of exposure, if occurring non-differentially between study arms, would bias the results from ResPECT towards the null hypothesis.

Key reasons for choosing a cluster-randomized design are (a) to increase compliance by equipping all members of a healthcare team with the same equipment and (b) to capture indirect effects of the intervention at the cluster-level, such as herd immunity [50].

A fully successful study would produce clinically relevant results that help clinician-leaders make reasoned decisions about protection of HCPs against occupationally acquired respiratory infections and prevention of spread within healthcare systems.

## Abbreviations

ARI, acute respiratory illness; CDC, centers for disease control and prevention; DSMB, data safety monitoring board; HAI, hemagglutination inhibition antibody; HCP, healthcare personnel; ILI, influenza like illness; LCRI, Laboratory confirmed respiratory illness; MM, medical mask; N95, N95 respirator; NIOSH, National Institute for Occupational Safety and Health; OSHA, Occupational Safety and Health Administration; PPE, occupational protective equipment; ResPECT, respiratory protection effectiveness clinical trial; RPD, respiratory protective devices; RT-PCR, reverse-transcriptase polymerase chain reaction; US, United States.
